# Genome-Wide Association Mapping of *bc-1* and *bc-u* Reveals Candidate Genes and New Adjustments to the Host-Pathogen Interaction for Resistance to *Bean Common Mosaic Necrosis Virus* in Common Bean

**DOI:** 10.3389/fpls.2021.699569

**Published:** 2021-06-29

**Authors:** Alvaro Soler-Garzón, Phillip E. McClean, Phillip N. Miklas

**Affiliations:** ^1^Irrigated Agriculture Research and Extension Center, Washington State University, Prosser, WA, United States; ^2^Department of Plant Sciences, North Dakota State University, Fargo, ND, United States; ^3^Grain Legume Genetics and Physiology Research Unit, United States Department of Agriculture - Agricultural Research Service (USDA-ARS), Prosser, WA, United States

**Keywords:** *Phaseolus vulgaris*, potyvirus, autophagy, hypersensitive response, transcription factors, marker-assisted selection, receptor-like kinases, synteny

## Abstract

Bean common mosaic necrosis virus (BCMNV) is a major disease in common bean (*Phaseolus vulgaris* L.). Host plant resistance is the primary disease control. We sought to identify candidate genes to better understand the host-pathogen interaction and develop tools for marker-assisted selection (MAS). A genome-wide association study (GWAS) approach using 182 lines from a race Durango Diversity Panel (DDP) challenged by BCMNV isolates NL-8 [Pathogroup (PG)-III] and NL-3 (PG-VI), and genotyped with 1.26 million *single-nucleotide polymorphisms* (SNPs), revealed significant peak regions on chromosomes Pv03 and Pv05, which correspond to *bc-1* and *bc-u* resistance gene loci, respectively. Three candidate genes were identified for NL-3 and NL-8 resistance. Side-by-side receptor-like protein kinases (RLKs), Phvul.003G038700 and Phvul.003G038800 were candidate genes for *bc-1*. These RLKs were orthologous to linked RLKs associated with virus resistance in soybean (*Glycine max*). A basic Leucine Zipper (bZIP) transcription factor protein is the candidate gene for *bc-u*. bZIP protein gene Phvul.005G124100 carries a unique non-synonymous mutation at codon 14 in the first exon (Pv05: 36,114,516 bases), resulting in a premature termination codon that causes a nonfunctional protein. SNP markers for *bc-1* and *bc-u* and new markers for *I* and *bc-3* genes were used to genotype the resistance genes underpinning BCMNV phenotypes in the DDP, host group (HG) differentials, and segregating F_3_ families. Results revealed major adjustments to the current host-pathogen interaction model: (i) there is only one resistance allele *bc-1* for the *Bc-1* locus, and differential expression of the allele is based on presence vs. absence of *bc-u*; (ii) *bc-1* exhibits dominance and incomplete dominance; (iii) *bc-1* alone confers resistance to NL-8; (iv) *bc-u* was absent from HGs 2, 4, 5, and 7 necessitating a new gene symbol *bc-u*^d^ to reflect this change; (v) *bc-u*^d^ alone delays susceptible symptoms, and when combined with *bc-1* enhanced resistance to NL-3; and (vi) *bc-u*^d^ is on Pv05, not Pv03 as previously thought. These candidate genes, markers, and adjustments to the host-pathogen interaction will facilitate breeding for resistance to BCMNV and related Bean common mosaic virus (BCMV) in common bean.

## Introduction

Bean common mosaic necrosis virus (BCMNV) and Bean common mosaic virus (BCMV) are related positive-stranded RNA viruses in the *Potyvirus* genus that infect common bean (*Phaseolus vulgaris* L.) worldwide. BCMNV and BCMV are transmitted by infected seeds and from plant to plant by several aphid species in a non-persistent manner. Seed-borne transmission plagues subsistence farmers and other growers who rely on planting their own “bin-run” seed. These viruses can cause greater than 80% yield loss in common bean production fields (Morales, 2003). The primary disease control is host plant resistance. Resistance to BCMNV and BCMV in the common bean is regulated by the dominant *I* gene and six recessive alleles (*bc-1*, *bc-1^2^*, *bc-2*, *bc-2^2^*, *bc-3*, and *bc-u*) distributed across four loci (Drijfhout, 1978). Strain diversity is classified into eight pathogroups (PG) based on interactions with 12 host group (HG) differential cultivars possessing different resistance gene combinations, PG I to VII referenced in Drijfhout et al. (1978) and PG VIII identified by Feng et al. (2015).

A few of the host resistance genes are well studied. The eIF4E [Eukaryotic translation initiation factor 4E; Phvul.006G168400 in Phytozome v13 info: P. vulgaris_G19833 v2.1 (Schmutz et al., 2014)] protein is a candidate gene for *bc-3* on chromosome Pv06 (Naderpour et al., 2010). eIF4E has a reported role in potyviral infection in other crops such as peas (*Pisum sativum*; Swisher Grimm and Porter, 2020), melon (*Cucumis melo*; Nieto et al., 2006), tomato (*Solanum lycopersicum*; Parrella et al., 2002), and pepper (*Capsicum annuum*; Ruffel et al., 2004). A missense *single-nucleotide polymorphism* (SNP) within eIF4E is diagnostic for *bc-3*, and a KASP marker designed for the SNP is used for marker-assisted selection (MAS) of *bc-3* in common bean (Hart and Griffiths, 2013). The *I* gene induces a temperature-independent hypersensitive response (HR) against BCMNV and either an immune or temperature-dependent HR response to BCMV strains (Drijfhout et al., 1978). The HR for *I* gene, without protection by any recessive genes (*bc-1*^2^, *bc-2*^2^, and *bc-3*), often results in the death of the plant. The *I* gene on Pv02 is linked with a cluster of seven NBS-LRR genes (Vallejos et al., 2006). An SNP marker just downstream from the NBS-LRR cluster is currently used for MAS of the *I* gene (Bello et al., 2014). Candidate genes for *bc-1*, *bc-2*, and *bc-u* have not been identified. However, *bc-1*^2^ is located on Pv03 (Miklas et al., 2000) and linked with a SCAR marker (SBD5) that is used for MAS of the gene (Vandemark and Miklas, 2005). Drijfhout (1978) characterized *bc-u* as a non-specific helper gene needed by the other five recessive *bc* genes to express resistance. *bc-u* was moderately linked (23 cM) with *bc-1*^2^ on Pv03 in a recombinant inbred population (Strausbaugh et al., 1999).

Candidate gene analysis of targeted traits is facilitated by new molecular tools for common bean, including millions of SNPs identified by resequencing genotypes and diversity panels (Cichy et al., 2015; Moghaddam et al., 2016; Lobaton et al., 2018; Wallace et al., 2018; Oladzad et al., 2019; Diaz et al., 2020; Wu et al., 2020), 6 and 12 K SNP BeadChip assays (Song et al., 2015; Miklas et al., 2020), and assembled bean genomes representing different gene pools, such as the Andean G19833 landrace (Chaucha Chuga; Schmutz et al., 2014) and “UI-111” cultivar for race Durango (DDP). These genomic tools contributed to candidate gene discovery for several traits in common bean, including the NAC candidate gene for *bgm-1* resistance to Bean golden yellow mosaic virus (BGYMV; Soler-Garzón et al., 2021), a bHLH transcription factor for the seed pigment *P* gene (McClean et al., 2018), and truncated CRINKLY4 kinase acting as a decoy to condition *Co-x* anthracnose resistance (Richard et al., 2021).

We sought to take advantage of a genome-wide association study (GWAS) approach to unravel the genetics of resistance to BCMNV in the Durango Diversity Panel (DDP). The objectives were to identify genomic regions associated with *bc-1* and *bc-u* resistance genes leading to candidate gene discovery and identification of polymorphisms to exploit for MAS.

## Materials and Methods

### Plant Materials

The DDP consists of 182 dry bean lines (cultivars, breeding lines, germplasm releases, and landraces) primarily from race Durango of the Middle American gene pool. Díaz and Blair (2006) grouped race Durango and race Jalisco genotypes because they were closely related as determined by microsatellite markers. Undoubtedly the DDP contains some Jalisco genotypes as well; however, we did not differentiate them. The panel was developed to provide a historical representation of pinto, great northern, pink, and small red market class genotypes released by North American breeding programs from the late 1930s to about 2010 (Supplementary Table S1).

Nine F_2_ populations were generated from crosses among DDP lines selected as parents according to their reactions to BCMNV inoculations and genotypes based on SNP markers linked to the *I*, *bc-1*, and *bc-u* genes (described below). Subsequently, the F_2_ populations were genotyped using the same SNP markers. About 241 F_2_ plants among the nine populations were genotyped. We selected and advanced 34 F_2_ plants (Supplementary Table S2) to further validate the segregation of the genes and linked markers. About 16 true-breeding F_2:3_ families were used as controls for each gene in a homozygous state, and the remaining 22 families had at least one gene in a heterozygous state. A total of 1,284 F_2:3_ plants were evaluated for BCMNV reaction and genotyped for the linked SNP markers (Supplementary Table S3).

### Evaluation of BCMNV Reaction

Screening of 182 DDP lines and 34 F_3_ families for BCMNV reaction using strains NL-8 D (henceforth NL-8) from PG 3 (PG-III) and NL-3 D (henceforth NL-3) from PG 6 (PG-VI) was conducted in the USDA-ARS greenhouses at Prosser (Washington, United States) under controlled conditions (22–28°C temperature range and 14-h photoperiod using artificial lights as necessary). The “D” suffix is indicative that NL-8 and NL-3 trace back to Drijfhout (1978) original isolates. Completed sequences for both isolates have been reported in previous phylogenetic studies (Larsen et al., 2005, 2011). Two sets of each DDP line and ~18 seeds per F_2:3_ family were grown in 9 cm^3^ pots containing three seeds each and a commercial potting mix (Sun Gro Horticulture, Bellevue, WA, United States). For each set, the primary leaves, when 80 to 100% fully expanded at approximately 10 days after planting (DAP), were mechanically inoculated (Drijfhout, 1978) with one of the BCMNV strains. Phenotypic data based on visual characterization and differential cultivars reactions were recorded at weekly intervals from 1 to 5 wpi (weeks post-inoculation).

The plant reactions to virus inoculations were categorized as, NS: no apparent symptoms on inoculated leaves and no systemic symptoms; VN: restricted vein necrosis on inoculated leaves, no systemic symptoms; VN^+^: restricted vein necrosis on inoculated leaves, with some small patches (10 mm^2^) of systemic restricted vein necrosis on upper trifoliolate leaves observed from 3 to 5 wpi; NLL: local necrotic lesions on inoculated leaves, no systemic symptoms; TN: lethal systemic top necrosis by 7–10 dpi (days post-inoculation), resulting in plant death; dTN: delayed TN beginning >11 dpi, most often resulting in plant death; M: leaf curling and plant stunting with severe systemic chlorotic mosaic symptoms; mM: mild systemic chlorotic mosaic symptoms; and dM: delayed severe systemic chlorotic mosaic symptoms observed 2–4 wpi. dM initiating at 2 wpi occurred in plants with NS, and at 4 wpi in plants with mM symptoms. The differential cultivars with reactions to BCMNV strains in parentheses (NL-8/NL-3): HG-1, “Sutter Pink” (M/M); HG-3, “Olathe” (NS/mM); HG-6, “Othello” (NS/NS); HG-8, “Widusa” (TN/TN); HG-9, “Top Crop” (VN/TN); HG-10, “Beryl” (VN/VN); and HG-11, “92US1006” (NLL/NLL) were used as controls.

### DNA Extraction and SNP Calling

DNA was extracted from each DDP member following the protocol described previously (Tobar-Piñón et al., 2021). Each DNA sample was sequenced at Hudson/Alpha Institute of Biotechnology (Huntsville, AL, United States) using Illumina technology to an average depth of 8X. WGS raw data have been deposited into the NCBI SRA database with accession BioProject PRJNA386820 (Supplementary Table S1).^2^

Reads of DDP were indexed, aligned, and sorted to the reference Andean G19833 *P. vulgaris* v2.1 genome sequence using BWA-MEM (Li, 2013) and Samtools (Li et al., 2009).^3^ A total of 11,518,066 variants [single nucleotide variants (SNVs), insertions or deletions (InDels), and short tandem repeats (STRs)], with at least 20 samples genotyped, were mapped to the 11 chromosomes and scaffolds of the G19833 v2.1 reference genome by next generation sequencing experience platform (NGSEP) pipeline (Perea et al., 2016; Lobaton et al., 2018). The maximum base quality score was set to 30, and the minimum base quality for reporting a variant was set to 40. All SNP markers detected with less than 50% missing values and a minor allele frequency (MAF) 0.05 were retained to perform imputation with the *ImputeVCF* module in NGSEP, which is a reimplementation of the Hidden Markov Model (HMM) applied in the package fastPHASE (Scheet and Stephens, 2006). Annotation of variants was performed using the command *Annotate* by NGSEP.

Genomic DNA in DDP and additional lines for SNP marker testing was isolated from 20 mg of leaf tissue collected from an individual plant for each line grown in the USDA-ARS greenhouses at Prosser, WA, United States, using a Qiagen DNeasy 96 Plant Kit (Hilden, Germany). For individual F_3_ plants, total DNA was extracted from four-leaf disks (approximately 30 mm^2^) according to the alkaline extraction method described by Xin et al. (2003) with modifications. Briefly, the method includes Buffer A (50 mM NaOH, 1% Tween® 20) to degrade cell walls and Buffer B (100 mM Tris-HCl, 1.7 mM EDTA pH 8) to neutralize DNA and bind proteins. About 200 μl of Buffer A were added, mixed by centrifuging at 1,500 rpm for 1 min, and incubated at 95°C for 10 min. About 120 μl of Buffer B was added, mixed by centrifuging at 1,500 rpm at room temperature for 5 min. A 1:7 dilution of the extracted DNA was placed in a 96-well plate with a final volume of 100 μl. At last, a 5 μl of DNA template dilution was used for PCR.

### Genome-Wide Association Study

Phenotypic and genotypic information was integrated for conducting GWAS based on a multi-locus random-SNP-effect mixed linear model (mrMLM) described by Wang et al. (2016) and implemented in the “mrMLM” R package (Wen et al., 2018). A kinship matrix was generated using the efficient mixed-model association (EMMA) algorithm implemented in the GAPIT R package (Lipka et al., 2012) with corrections for kinship and population structure. Five PCs generated from GAPIT were included as covariates. The Bonferroni test was implemented to control the experiment-wise type *I* error rate at 0.05. GWAS results were plotted using CMPlot v3.62 (Yin, 2016), and IntAssoPlot v3 (He et al., 2020) was used to represent regional and single gene-based marker-trait associations graphically.

### Candidate Gene Identification and Development of SNP Markers

Candidate genes located within the associated genomic regions were identified by aligning with the G19833 reference transcriptome v2.1, perusing available scientific literature, and synteny comparison with soybean (*Glycine max*).^4^ Exon sequences of each *bc-1* and *bc-u* candidate gene were amplified across select genotypes (Supplementary Table S4) using PCR primers designed with Primer3 software (Koressaar and Remm, 2007; Untergasser et al., 2012). Two standard PCRs, for each sample, were replicated in a volume of 25 μl contained 1.8 mM MgCl_2_, 0.4 mM of the dNTPs mix (Promega™, Madison, WI), 0.25 μM of each primer (forward and reverse), 25 ng of genomic DNA, and 1 unit Taq DNA polymerase (Promega), in 1 × PCR buffer (Promega), under the following amplification conditions: heating for 2 min at 95°C, followed by 38 cycles at 94°C for 20 s, annealing specific temperature for each primer set for 30 s and 72°C for 90 s, and a final extension at 72°C for 5 min. All the PCR amplifications were performed in a PCR Eppendorf Mastercycler (Eppendorf AG, Hamburg, Germany). PCR fragments were visualized by gel electrophoresis on 2% (w+v) agarose. The PCR fragments were purified and Sanger sequenced by Eurofins MWG Operon (Louisville, KY, United States). Sequence trimming, alignment, and polymorphism discovery were performed with Geneious 9.1.2 software (Kearse et al., 2012).

A set of allele-specific primers were designed according to Wang et al. (2005) for polymorphic SNPs identified in coding regions of the sequenced *bc-1* and *bc-u* candidate genes using the Primer3 software (Koressaar and Remm, 2007; Untergasser et al., 2012). Similar primers were designed for one missense SNP detected by GWAS, linked to the *I* gene. These markers were named S03_4203361 (*bc-1* gene), Pvbzip1_A_C (*bc-u* gene), and S02_48908259 (*I* gene). Fragments were amplified by PCR on an Eppendorf Mastercycler using a PCR volume of 20 μl containing 1.5 mM MgCl_2_, 0.2 mM of the dNTPs mix (Promega), 0.15 μM of each primer (two allele-specific forward primers and the common reverse primer), 1X EvaGreen™ (Biotium, Fremont, CA), 20 ng of genomic DNA, 1X Taq buffer, and 0.1 μl *Taq*1 polymerase (Promega) under the following thermal profile: an initial denaturation step at 94°C for 2 min, then 38 cycles of denaturation at 92°C for 20 s, annealing for 20 s (the temperature was specific to each primer trio), and extension at 72°C for 20 s, and a final extension at 72°C for 5 min. Melting point analysis for allele determination of the template DNA was performed with a fluorescence-detecting thermocycler (LightCycler™ 4890 Instrument II, Roche, Basal Switzerland) with EvaGreen™ fluorescent dye (Biotium). Fluorescent detection was performed for 1 min at 95°C and the melting curve step ramping was performed from 65 to 95°C in increments of 1°C every 20 s. Screening for the *bc-3* gene in DDP lines was also performed using the primer assay designed by Hart and Griffiths (2013), which we modified for the temperature melting (Tm)-shift assay, SNP genotyping method (Wang et al., 2005). The same PCR amplification protocol described above was used, except for modifying the annealing temperature to 69°C (Table 1).

**Table 1:** Primers were used to generate markers for *bc-1*, *bc-u*^d^, *I*, and *bc-3* genes conditioning resistance to Bean common mosaic necrosis virus (BCMNV) and Bean common mosaic virus (BCMV).

BCMNV resistance genes	ID marker		Sequence	Ta (°C)	Chr	Position (G19833v2.1)	Sense	Allele Resistant	Allele Susceptible
*bc-1*	S03_4203361	*Fa*	gcgggcTGGTCAGTTTGTCTTCCCTAACT	60	Pv03	4,203,361	+	T	A
		*R*	TGCAGAAGAGCTCAACTCGAAG						
		*Fb*	gcgggcagggcggcGGTCAGTTTGTCTTCCCTAACA						
*bc-u*^d^	Pvbzip1_A_C	*Fa*	gcgggcTAGGAGAACTTGGTTTGTCTGAGTA	66	Pv05	36,114,516	+	A	C
		*R*	GCACTCCATAAGGGATGTGGT						
		*Fb*	gcgggcagggcggcGGAGAACTTGGTTTGTCTGAGTC						
*I*	*S02_48908259*	*Fa*	gcgggcCAAAGTGCTAGAGGCATGATCA	58	Pv02	48,908,259	+	T	A
		*R*	TGGTTATCATTCATTGTGAAGTCAATG						
		*Fb*	gcgggcagggcggcCAAAGTGCTAGAGGCATGATCT						
*bc-3*	PveIF4E^1,3,4^ _PveIF4E^2^	*Fa*	gcgggcCAATCTTATGCTTGAAGCAGTGAAAGT	69	Pv06	27,204,768	−	G	A
		*R*	ATTTACAATAACATTCACCACCCGAGCAA						
		*Fb*	gcgggcagggcggcAATCTTATGCTTGAAGCAGTGAAAGC						

## Results

### Bean Common Mosaic Necrosis Virus Screening

A total of 182 DDP lines were evaluated for reaction to BCMNV strains NL-3 (PG-VI) and NL-8 (PG-III) under greenhouse conditions. The DDP lines could be separated into two major groups based on M, dM, and mM vs. TN, VN, VN^+^, and NLL symptoms indicating absence (96 lines) and presence of the dominant *I* gene (61 lines), respectively (Table 2; Supplementary Table S5). The *I* gene marker assayed across the DDP lines verified these groupings, except for AC Polaris with mM symptoms, which lacked the *I* gene but possessed the marker. Based on symptoms, 25 DDP lines did not fit the above groups due to segregating reactions (seven lines), NS due to the presence of the *bc-3* gene (six lines) as verified by the PveIF4E^1,3,4^_PveIF4E^2^ marker, or NS due to the presence of the *bc-2^2^* allele based on the published information. Comparing the reactions to both BCMNV strains resulted in nine categories (Table 2) with mM to NL-3 and NS to NL-8 (26.4% of the lines) and TN to both strains (17.0%) representing the two largest categories, and NLL to both strains (1.1%) was the category with the fewest number of lines.

**Table 2:** Phenotypic reactions to BCMNV isolates NL-8 and NL-3observed in Durango Diversity Panel (DDP; 182 lines), classified into two major groups: recessive *I* gene vs. dominant *I* gene, with four and five phenotype sub-categories, respectively.

Group	DDP lines no.	NL-8	NL-3	^a^Allelic combination detected	Lines no.	Lines %
*i* allele	96 (four categories)	No Symptoms	mild Mosaic	*bc-1bc-1*/*bc-u*^d^*bc-u*^d^	48	26.4
		Mosaic	Mosaic	*No genes present*	17	9.3
		delayed Mosaic	Mosaic	*bc-u*^d^*bc-u*^d^	16	8.8
		No Symptoms	Mosaic	*bc-1bc-1*	15	8.2
*I* allele	61 (five categories)	Top Necrosis	Top Necrosis	*II*	31	17.0
		delayed Top Necrosis	Top Necrosis	*II*/*bc-u*^d^*bc-u*^d^	11	6.0
		Vein Necrosis	Vein Necrosis	*II* / *bc-1bc-1* / *bc-u*^d^*bc-u*^d^	9	4.9
		Vein Necrosis	Top Necrosis	*II / bc-1bc-1*	8	4.4
		Necrotic Local Lesion	Necrotic Local Lesion	^b^*II*/*bc-u*^d^*bc-u*^d^/*bc-2*^2^*bc-2*^2^	2	1.1
Misc.	25	No Symptoms	No Symptoms	^b^*bc-2*^2^ present	12	6.6
		No Symptoms	No Symptoms	*bc-3* present	6	3.3
		Segregating	Segregating	Segregating	7	3.8

Twenty-five miscellaneous lines were not included in the analyses. Subsequently, the DDP was assayed with SNP markers to detect bc-1, bc-u^d^, I, and bc-3 genes.

a Presence of the *bc-1*, *bc-u*^d^, *I*, and *bc-3* genes based on temperature melting (Tm)-shift SNP marker assays (as shown in Table 1).

b Presence of the *bc-2*^2^ allele based on published information.

### Genome-Wide Association Analysis

**Figure 1:**
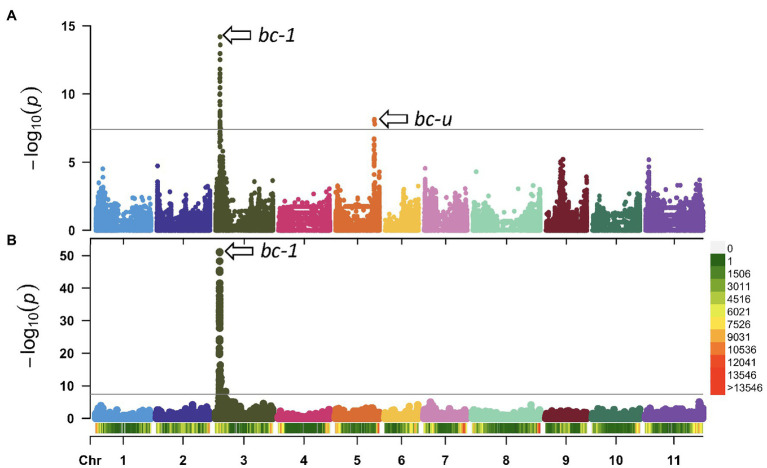
Manhattan plots of common mosaic necrosis virus (BCMNV) resistance in DDP. The gray horizontal line represents the genome-wide significance threshold of *p* = 3.98 × 10^−8^. **(A)** genome-wide association study (GWAS) using lines exhibiting mild mosaic (mM) and lines with mosaic (M) symptoms inoculated with NL-3 strain, two strong associations on Pv03 (*bc-1*) and Pv05 (*bc-u*^d^). **(B)** GWAS using lines with no symptoms (NS) compared with lines showing mosaic (M) symptoms against NL-8 strain, peak detected on Pv03 (*bc-1*). Bottom: Density bar (color-coded) showing genome distribution of 1,269,044 biallelic SNPs within 1 Mb window size in the DDP.

Genome-wide association studies were performed to identify genetic regions associated with BCMNV resistance using the filtered 1,269,044 biallelic SNPs and the DDP lines homozygous recessive *i* for the *I* gene (Table 2). The first GWAS was conducted with 48 DDP lines with partial mM resistance and 48 lines with susceptible M reactions to NL-3 strain. A second GWAS was performed with 63 DDP lines with resistant NS and 33 lines with susceptible M or dM reactions to NL-8. The Manhattan plots revealed two different loci associated with partial resistance to NL-3 and one locus for resistance to NL-8 strain (Figures 1A,B). These loci, exceeding the Bonferroni-corrected *α* = 0.05 threshold (*p* = 3.9E-08), were mapped to Pv03: 4.1 Mb and Pv05: 36.1 Mb.

The locus on chromosome Pv03, detected by both GWAS populations, is associated with the recessive *bc-1*^2^ allele (Drijfhout, 1978; Strausbaugh et al., 1999) linked to the SDB5 SCAR marker (Miklas et al., 2000) located on Pv03 at 4,204,238 bp in the Andean G19833 v2.1 reference genome. The second locus associated with resistance to NL-3 on chromosome Pv05 is putatively the *bc-u* recessive gene. This Pv05 location is inconsistent with the Pv03 location for *bc-u* observed by Strausbaugh et al. (1999). For this putative interaction model, the *bc-1*^2^ and *bc-u* gene combination conditions partial mM resistance (Kelly, 1997) to NL-3, and *bc-1*^2^ alone conditions NS resistance to NL-8. The *bc-1*^2^ and *bc-u* combination for HG-3 fits the model of Drijfhout (1978) with *bc-u* acting as complementary strain-unspecific helper gene necessary for the expression of *bc-1*^2^ resistance to BCMNV or BCMV strains. However, *bc-1*^2^ mediated resistance to NL-8 strain in the absence of *bc-u* does not fit the model of Drijfhout. His model for HG-2 has *bc-1* and *bc-u* contributing NS resistance to NL-8. For our model going forward, *bc-1* and *bc-1*^2^ are the same alleles, and the differential reaction observed with NL-3 and NL-8 strains is due to the presence vs. absence of *bc-u*.

**Figure 2:**
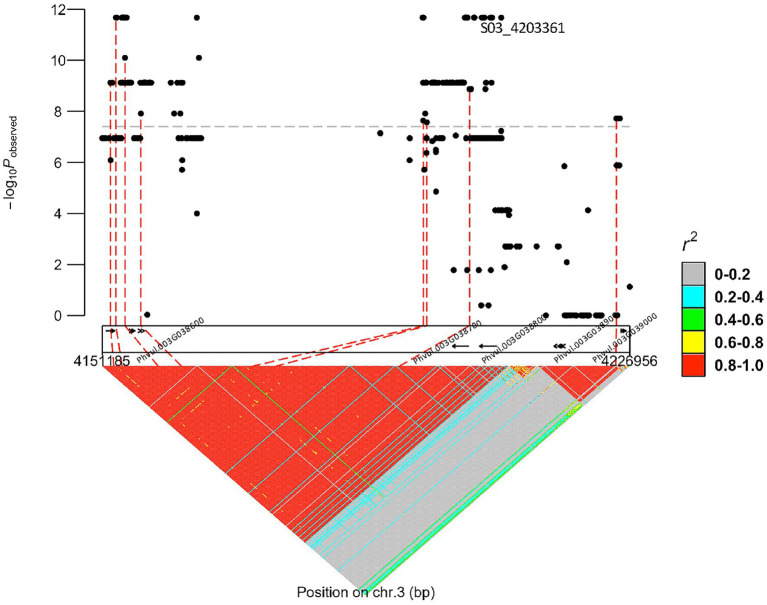
*bc-1* regional marker-trait associations plot depicting the receptor-like protein kinases (RLK) candidate genes (arrows) for *bc-1* inside the dotted box rectangle.

Based on the whole-genome sequences for the DDP, there were 29 gene models spanning the peak region (3,861,624 bp–4,364,674 bp, determined by the significance threshold in Figure 1) on Pv03 where *bc-1* maps. About 19 models possessed 225 SNVs, 16 InDels, and seven STRs in coding regions. Two genes in high linkage disequilibrium (LD), Phvul.003G038700 and Phvul.003G038800, were identified from a literature survey and synteny block analysis as candidate genes for *bc-1* (Figure 2). Synteny between common bean and soybean [*G. max* (L.) Merrill] revealed that these two *bc-1* candidate genes were orthologous to soybean Glyma.02g121900 and Glyma.02g122000 gene models (Table 3). These soybean gene models were identified in separate studies to be candidate genes for resistance to the Tobacco ringspot virus (TRSV; Chang et al., 2016) and Soybean mosaic virus (SMV; Maroof et al., 2010; Ilut et al., 2015). These candidate genes encode protein receptor-like protein kinases (RLK; Chang et al., 2016) with a potential role in delaying virus replication and ﻿affecting the systemic spread of BCMV (Maroof et al., 2010; Feng et al., 2017).

**Table 3:** Synteny analysis between common bean and soybean comparing *bc-1* and *Rsv4* candidate genes (bold type), respectively.

*Phaseolus vulgaris* G19833 v2.1	Chromosome location	*Glycine max* Wm82.a2.v1	BLASTp Phytozone E-value; Identity	Annotation
Phvul.003G038300	Pv03: 4,023,174–4,028,058	Glyma.02G122700	0.0; 66.9%	18S Pre-Ribosomal assembly protein GAR2-related protein
Phvul.003G038400	Pv03: 4,121,926–4,126,390	Glyma.02g122500	0.0; 94.2%	ACT domain repeat 5
Phvul.003G038500	Pv03: 4,139,554–4,140,060	Glyma.02g122400	8.0E-40; 81.7%	Putative unknown protein
Phvul.003G038600	Pv03: 4,151,102–4,157,419	Glyma.02g122200	0.0; 70.2%	Chaperone DNAJ-domain superfamily protein
**Phvul.003G038700**	**Pv03: 4,201,273–4,204,095**	**Glyma.02G121900**	**0.0; 75.9%**	**Receptor-Like Protein Kinase**
**Phvul.003G038800**	**Pv03: 4,205,380–4,207,902**	**Glyma.02G122000**	**0.0; 78.9%**	**Receptor-Like Protein Kinase**
Phvul.003G038900	Pv03: 4,215,776–4,217,396	Glyma.02g121800	1.2E-154; 86.3%	Adenine nucleotide alpha hydrolases-like superfamily protein
Phvul.003G039000	Pv03: 4,225,490–4,229,239	^a^Glyma.08G302400 ^a^Glyma.13G178100	0.0; 76.0% 0.0; 81.5%	MIF4G domain-containing protein/MA3 domain-containing protein
Phvul.003G039100	Pv03: 4,232,478–4,234,069	Glyma.02g121700	8.7E-28; 44.6%	RING/U-box Zinc finger, C3HC4 type protein

a These orthologous genes in soybean for Phvul.003G039000 are on different chromosomes.

Phvul.003G039000, which encodes a MIF4G (middle domain of the eIF4G) domain-containing protein, is another putative candidate gene for *bc-1* (Table 3). MIF4G conditions resistance to Rice yellow mottle virus (RYMV) in *Oryza sativa* (Albar et al., 2006). MIF4G is absent from the orthologous Gm02 region, so we conducted a protein–protein BLAST of the Phvul.003G039000 MIF4G protein, against the *G. max*, Wm82.a2.v1 reference genome. Similar protein sequence identity alignments on chromosomes Gm08 (Glyma.08G302400) and Gm13 (Glyma.13G178100; *E*-value = 0; identity = 76–81.5%, respectively) were obtained from the BLAST search. Glyma.13G178100 is 0.66 Mb upstream from the *Rsv1* gene conditioning resistance to SMV and BCMV (Wu et al., 2019), which increases support of MIF4G (Phvul.003G039000) as a candidate gene for potyvirus resistance in common bean.

**Figure 3:**
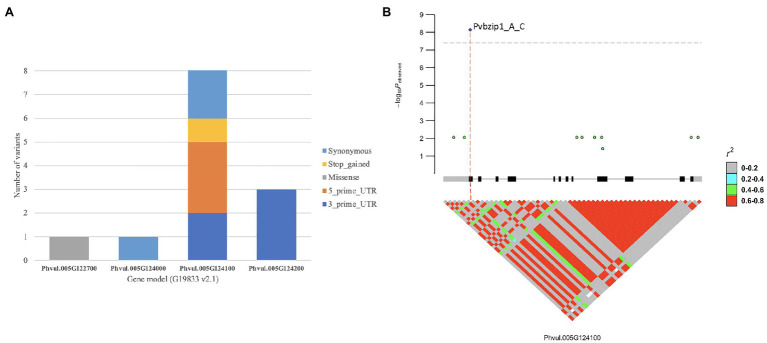
Candidate genes in *bc-u* region detected by GWAS: **(A)** coding variants distributed across gene models for the *bc-u*^d^ gene region, and **(B)** Phvul.005G124100 basic Leucine Zipper (bZIP) gene-based marker-trait associations plot.

There were 15 gene models spanning the peak region (Pv05: 36,000,235–36,127,721 bases) for *bc-u*, with 289 SNVs, 58 InDels, and 18 STRs (Table 4). Coding variants were discovered in Phvul.005G122700 (one missense variant), Phvul.005G124000 (one synonymous variant), Phvul.005G124100 [two 3′ UTR (Un-Translated Regions), three 5′ UTR, one stop-gain, and three synonymous variants], and Phvul.005G124200 (three 3′ UTR variants; Figure 3A). No variants were found in Phvul.005G12320, Phvul.005G123600, and Phvul.005G123900 gene models. GWAS results revealed the stop-gain mutation in Phvul.005G124100 as the highest associated SNP for the *bc-u* gene (Figure 3B). A stop-gain mutation results in a premature termination codon that causes a shortening and likely reduced protein function. The Phvul.005G124100 gene model contains basic Leucine Zipper (bZIP) transcription factor and Delay of Germination 1 (DOG1) domains. bZIP transcription factors have been related to pathogen infection, stress signaling, seed maturation, and flower development in different crop species (Kaminaka et al., 2006; Liao et al., 2008; Alves et al., 2013; Gao et al., 2014). The *Arabidopsis* bZIP ortholog TGA9 (AT1G08320; BLASTp *E*-value = 7.74E-180; identity = 60%) is an active regulatory factor in autophagy, a physiological response in host-pathogen interactions (Wang et al., 2020). Consequently, of the 15 gene models, Phvul.005G124100 was chosen as the most likely candidate gene for *bc-u*.

**Table 4:** Candidate gene (bold type) for the *bc-u*^d^ gene region.

Phaseolus vulgaris G19833 v2.1	Chromosome location	Annotation
Phvul.005G122700	Pv05: 36,000,235–36,000,933	Senescence regulator
Phvul.005G122800	Pv05: 36,006,618–36,009,521	FAMILY NOT NAMED
Phvul.005G122900	Pv05: 36,012,213–36,012,827	Cupin domain
Phvul.005G123100	Pv05: 36,031,013–36,035,680	N-carbamoylputrescine amidase (aguB)
Phvul.005G123200	Pv05: 36,039,621–36,040,692	Fantastic Four meristem regulator (FAF)
Phvul.005G123300	Pv05: 36,043,640–36,047,570	Rho GTPase activating protein with PAK-box / P21-Rho-binding domain
Phvul.005G123400	Pv05: 36,053,256–36,055,519	Calcium-binding EF-hand family protein
Phvul.005G123600	Pv05: 36,059,079–36,062,903	Glycosyl hydrolase superfamily protein
Phvul.005G123500	Pv05: 36,060,209–36,060,364	No annotation
Phvul.005G123700	Pv05: 36,074,164–36,076,099	Ribosomal protein L1p/L10e family
Phvul.005G123800	Pv05: 36,087,411–36,087,806	No annotation
Phvul.005G123900	Pv05: 36,094,573–36,097,466	Homeodomain-like superfamily protein/MYB-CC type trans-factor, LHEQLE motif
Phvul.005G124000	Pv05: 36,104,915–36,107,979	No annotation
**Phvul.005G124100**	**Pv05: 36,113,780–36,120,803**	**bZIP transcription factor family protein**
Phvul.005G124200	Pv05: 36,125,362–36,127,721	Basic-leucine zipper (bZIP) transcription factor family protein

**Figure 4:**
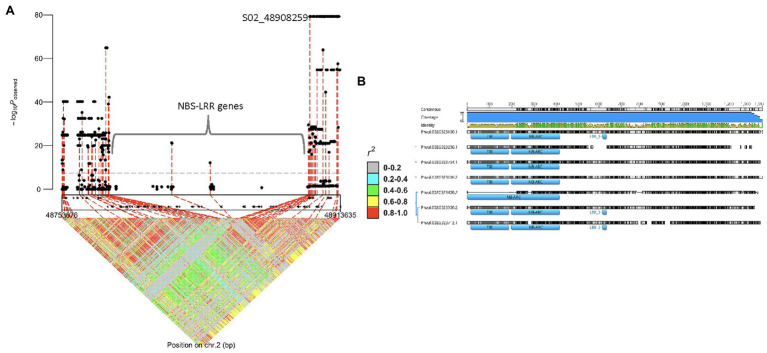
Regional association plot displaying GWAS results and candidate genes for *I* gene: **(A)** *I* gene regional marker-trait associations and linkage disequilibrium plots, and **(B)** alignment of the amino acid sequences of NBS-LRR cluster proteins.

A separate GWAS, using DDP lines with (96 lines) and without (61 lines) *I* gene, detected a significant interval of 48.0–49.4 Mb on Pv02 as expected (Figure 4A). The 48.8 peak position was near the cluster of seven NBS-LRR (R gene motifs) gene models, from 48,786,657 to 48,895,789 base pairs, previously reported to have a putative association with the *I* gene (Vallejos et al., 2006; Bello et al., 2014). The high protein sequence identity ranging from 53.4 to 82.0% among the seven NBS-LRR genes limited the marker saturation for this region (Figure 4B). Genomic regions with repetitive DNA present a technical challenge for calling high-quality variants for use in GWAS and subsequent candidate gene analysis (Treangen and Salzberg, 2012). The GWAS results identified one missense SNP with a high *p*-value (*p* = 5.7E-80) on Pv02: 48,908,259 bases within the Phvul.002G324100 gene model encoding a PLATZ (Plant AT-rich sequence and zinc-binding) transcription factor family protein. This SNP is in high LD with the SNP identified by Bello et al. (2014), with both SNPs exhibiting 99.4% accuracy for detecting the *I* gene in the DDP. AC-Polaris expressing mild mosaic (mM) symptoms to NL-3, indicating the absence of *I* gene, possessed both SNPs (false positive), which was the only mismatch for these *I* gene-linked markers in the DDP.

### Sequencing Candidate Genes

Twenty-five lines representing different BCMNV/BCMV HGs were chosen for targeted sequencing for the ORF of *bc-1* and *bc-u* candidate genes (Supplementary Tables S6–S8; Supplementary Figures S1 and S2). For these lines, sequences of *bc-1* candidate genes aligned to G19833 genome identified 22 missense SNPs, 10 synonymous SNPs, and one InDel in-frame insertion for Phvul.003G038700, and 27 missense SNPs, 13 synonymous SNPs, one in-frame InDel insertion and two in-frame InDel deletion variants for Phvul.003G038800 gene. No introns were detected for either of the RLK *bc-1* candidate genes. Variants found in Phvul.003G038700 and Phvul.003G038800 were not polymorphic between lines reported as *bc-1* and *bc-1*^2^ in previous studies (Drijfhout, 1978; Strausbaugh et al., 1999). This result and segregation analyses below further support *bc-1* and *bc-1^2^* as the same allele, which exhibits a differential effect based on the presence vs. absence of *bc-u*^d^.

For the ORF sequences of candidate gene Phvul.003G39000 MIF4G domain-containing protein, there were 16 missense SNPs, 12 synonymous SNPs, and two InDels (one in-frame InDel deletion and another splice region variant) found. These variants were grouped in six haplotypes, of which two haplotypes (Hap1 and Hap2) were identified in lines with *bc-1* gene and Andean background, and four haplotypes (Hap3–Hap6) in lines with Durango background, but only Hap3 possessed the *bc-1* gene (Supplementary Table S7).

The sequence of Phvul.005G124100 (bZIP), a candidate gene for *bc-u*, was analyzed by BLASTn to the G19833 genome (Supplementary Table S8). Multiple mutations were detected, including one stop-gain SNP in the first exon, two synonymous SNPs in the ninth exon, and one synonymous SNP in the twelfth exon. The protein sequence for the stop-gain mutation (Pv05:36,114,516 bases) exhibited a nonfunctional protein containing 14 amino acids in the Common Red landrace (Supplementary Figure S1). The unique nonsynonymous mutation comprises a single base transversion at codon 14 in the first exon. A preliminary gene symbol, *bc-u*^d^, is proposed for this mutation Pvbzip1_A_C due to its high frequency (55%) in the DDP. The “d” superscript signifies the *bc-u* allele of race Durango origin.

### Temperature Melting-Shift Genotyping With Candidate SNP Markers

Single-nucleotide polymorphisms identified in coding regions of candidate genes for *bc-1* and *bc-u*^d^ were converted to Tm-shift assays (Wang et al., 2005). S03_420336 is a missense SNP in the RLK (Phvul.003G038700) candidate gene for *bc-1* (Supplementary Figure S2). Due to the high number of missense variants found in the candidate genes for *bc-1*, this SNP was chosen based on primer design parameters such as % GC content and self-complementary score. Pvbzip1_A_C is the unique nonsynonymous SNP variant found in the Phvul.005G124100 candidate for the *bc-u*^d^ gene. Additional Tm-shift assays were developed for SNPs to track the *I* and *bc-3* genes. S02_48908259 is a missense SNP in Phvul.002G324100 PLATZ transcription factor, which is ~67 kb downstream from the NBS-LRR gene cluster and tightly linked with the *I* gene. The missense SNP at 27,204,768 bases (G19833 v2.1) in Phvul.006G168400 was used for the PveIF4E^1,3,4^_PveIF4E^2^ assay for *bc-3* gene developed by Hart and Griffiths (2013). These Tm-shift assay markers (Supplementary Figures S3–S6) were used for genotyping the DDP, and progeny of the F_2_ and F_3_ populations for the presence of *bc-1*, *bc-u*^d^, *I*, and *bc-3* to ascertain the resistance genes underpinning the phenotypic responses to BCMNV.

### DDP Assays

The nine phenotypic categories for reaction to NL-8 and NL-3 strains in the DDP had distinct fixed genotypes for the *I*, *bc-1*, and *bc-u*^d^ markers (Table 2; Supplementary Table S5). DDP lines with susceptible M symptoms to both strains lacked the resistance marker allele for all three resistance genes. Lines with dM to NL-8 and M to NL-3 had only the *bc-u*^d^ resistance allele, indicating *bc-u*^d^ alone had a minor effect in delaying, by about 7d, susceptible reactions to some BCMNV strains. DDP lines with only the *bc-1* resistance allele were resistant (NS) to NL-8 and susceptible (M) to NL-3. DDP lines with both the *bc-1* and *bc-u*^d^ resistance alleles were similarly (NS) resistant to NL-8 but had partial resistance (mM) to NL-3.

Lines with resistant *I* and *bc-u*^d^ alleles exhibited a similar effect of dTN to NL-8 strain by about 1 week, whereas these same lines had TN to NL-3. DDP lines with resistance *I* and *bc-1* alleles exhibited resistant VN to NL-8 and susceptible TN to NL-3. However, lines with the *bc-u*^d^ resistance allele in addition to *I* + *bc-1* alleles expressed VN to both strains, which shows a critical role for *bc-u*^d^ in protecting *I* + *bc-1* lines against NL-3 strain. DDP lines with TN to both strains possessed just the *I* gene unprotected by any of the recessive “*bc*” genes.

### Host Group Assays

Differential genotypes distributed across the 12 HGs for BCMNV/BCMV and one unassigned genotype UI-129 were assayed for the *I*, *bc-1*, and *bc-u*^d^ linked SNP markers (Table 5). All HGs (HG-2, HG-3, HG-5, HG-9, and HG-10) reported in previous studies with *bc-1* or *bc-1*^2^ carried the *bc-1* linked S03_4203361 SNP and the SBD5 SCAR markers. The SBD5 marker, located on Pv03 (4,204,236–4,205,565 bases), is in high LD with the S03_4203361 SNP in the DDP lines (data not shown). HG-1 differentials, not expected to possess *bc-1*, were mixed for the presence of *bc-1* linked markers. For the HG-2, HG-5, and HG-9 genotypes previously reported to possess *bc-1*, none had the *bc-u*^d^ gene marker, whereas most of HG-3 and HG-10 genotypes, previously reported to possess *bc-1*^2^, also possessed *bc-u*^d^, with few exceptions. The Andean lines Redlands Greenleaf B (HG-3) and Amanda (HG-10) did not possess *bc-u*^d^.

**Table 5:** Single-nucleotide polymorphism genotyping of BCMNV and BCMV host differential genotypes.

Line	Genepool	Host Group	Proposed Resistance Genotype	^a^NL8	^a^NL-3	^b^S02_48908259	^b^S03_4203361	^c^SBD5	^b^Pvbzip1_A_C	^d^PveIF4E^1,3,4^_PveIF4E^2^
						Pv02: 48,908,259	Pv03: 4,203,361	Pv03: 4,204,238	Pv05: 36,114,516	Pv06: 27,204,768
						*I* gene	*bc-1*	*bc-1*	*bc-u*^d^	*bc-3*
Dubbele Witte	Mesoamerican	1	none	M	M	−	−	−	−	−
Sutter Pink	Durango	1	none	M	M	−	−	−	−	−
Bountiful	Andean	1	^e^*bc-1*	M	M	−	+	+	−	−
Stringless Green Refugee (SGR)	Andean	1	^e^*bc-1*	M	M	−	+	+	−	−
Poncho (DDP041)	Durango	1	*bc-u*^d^	dM	M	−	−	−	+	−
Imuna	Andean	2	*bc-1*	NS	M	−	+	+	−	−
Redlands Greenleaf C (RGC)	Andean	2	*bc-1*	NS	M	−	+	+	−	−
UI-59 (DDP076)	Durango	3	*bc-1*, *bc-u*^d^	NS	mM	−	+	+	+	−
Olathe	Durango	3	*bc-1*, *bc-u*^d^	NS	mM	−	+	+	+	−
Common Red (DDP067)	Durango	3	*bc-1*, *bc-u*^d^	NS	mM	−	+	+	+	−
Redlands Greenleaf B (RGB)	Andean	3	^f^*bc-1*, *bc-?*	NS	mM	−	+	+	−	−
Michelite 62	Mesoamerican	4	*bc-2*, *bc-?*	M	M	−	−	−	−	−
Sanilac	Mesoamerican	4	*bc-2*, *bc-?*	M	M	−	−	−	−	−
UI-111 (DDP077)	Durango	4	*bc-2*, *bc-?*	M	M	−	−	−	−	−
UI-34	Durango	4	*bc-2*, *bc-?*	M	M	−	−	−	−	−
UI-114 (DDP078)	Durango	5	^f^*bc-1*, *bc-2*, *bc-?*	NS	M	−	+	+	−	−
Othello (DDP109)	Durango	6	*bc-1*, *bc-2*^2^, *bc-u*^d^	NS	NS	−	+	+	+	−
GN-31	Durango	6	*bc-1*, *bc-2*^2^, *bc-u*^d^	NS	NS	−	+	+	+	−
UI-129	Durango	X	^f^*bc-1*, *bc-2*, *bc-?*	NS	M	−	+	+	−	−
IVT-7214	Mesoamerican	7	^f^*bc-2*, *bc-3*, *bc-?*	NS	NS	−	−	−	−	+
Black Turtle I	Mesoamerican	8	*I*	TN	TN	+	−	−	−	−
Widusa	Andean	8	*I*	TN	TN	+	−	−	−	−
Gemini (DDP152)	Durango	8	*I*, *bc-u*^d^	dTN	TN	+	−	−	+	−
Jubila	Andean	9a	*I*, *bc-1*	VN	VN^+^	+	+	+	−	−
Topcrop	Andean	9b	*I*, *bc-1*	VN	TN	+	+	+	−	−
Amanda	Andean	10	^f^*I*, *bc-1*, *bc-?*	VN	VN	+	+	+	−	−
Beryl (DDP055)	Durango	10	*I*, *bc-1*, *bc-u*^d^	VN	VN	+	+	+	+	−
92US-1006 (DDP108)	Durango	11	*I*, *bc-2*^2^, *bc-u*^d^	LLN	LLN	+	−	−	+	−
IVT-7223	Mesoamerican	11	*I*, *bc-2*^2^, *bc-u*^d^	LLN	LLN	+	−	−	+	−
TARS-VR-8S	Mesoamerican	12	*I*, *bc-3*, *bc-u*^d^	NS	NS	+	−	−	+	+
USCR-7	Andean	12	*I*, *bc-1*, *bc-3*	NS	NS	+	+	+	−	+
USLK-3	Andean	12	*I*, *bc-1*, *bc-3*, *bc-u*^d^	NS	NS	+	+	+	+	+
Raven	Mesoamerican	12	*I*, *bc-3*	NS	NS	+	−	−	−	+

a NS, no symptoms; VN, vein necrosis; VN^+^, vein necrosis on upper trifoliolate leaves; NLL, local necrotic lesions; TN, top necrosis; dTN, delayed top necrosis; M, mosaic; mM, mild mosaic; dM, delayed mosaic.

b SNP markers developed in this study.

c SCAR marker developed by Miklas et al. (2000).

d SNP variant identified by Hart and Griffiths (2013) but modified to the Tm-shift SNP genotyping method.

e *bc-1* for these HG-1 cultivars was unexpected and will require further investigation.

f *bc-?* indicates a helper gene other than *bc-u*^d^ and work characterizing these other genes is in progress.

A pattern for the presence of *bc-u*^d^ and its effect when present can be hypothesized for race Durango lines across the host differentials in Table 5 and Supplementary Table S8. Briefly, *bc-u*^d^ is present in race Durango lines in HG-3, HG-6, HG-10, and HG-11. All these HGs possess the *bc-1*^2^ (formerly) or *bc-2*^2^ alleles. Conversely, *bc-u*^d^ is absent in HG-2, HG-4, and HG-9, which possess the alternative *bc-1* and *bc-2* alleles.

### Allelism Test in F_2:3_ Families

#### Fixed Genotypes

The observed segregations for phenotypic reactions to BCMNV strains across F_2:3_ families from F_2_ plants with the same genotypes for *I*, *bc-1*, and *bc-u*^d^ based on linked markers are summarized in Table 6 and presented in greater detail in Supplementary Tables S9 and S10. For even more detail, the genotype and phenotype for each F_3_ plant are in Supplementary Table S3. Each resistant gene-linked SNP marker fit expected codominant 1:2:1 ratio in all segregating populations and families (*df* = 2.0, *p* ≤ 0.05: 5.99). Accordingly, F_2_ individuals homozygous for the gene-linked markers bred true in the F_3_ generation. The true-breeding F_2:3_ families for one or more markers matched the phenotypes of the parents and other DDP lines with the same marker genotypes (Supplementary Table S5). For example, F_2:3_ plants or families fixed for *I* + *bc-1* linked markers exhibited VN to NL-8 and TN to NL-3 (e.g., parental lines “Orion” and “Powderhorn”); *I* + *bc-u*^d^ exhibited dTN to NL-8 and TN to NL-3 (“Gemini” and “Lariat”); and finally, *I* + *bc-1* + *bc-u*^d^ exhibited VN to both strains (“Beryl” and “Kodiak”). It was previously thought that the VN response to NL-3 strain was conditioned by the *I* + *bc-1*^2^ combination (Drijfhout, 1978; Kelly, 1997; Miklas et al., 2000), whereas the results indicate *bc-1* requires *bc-u*^d^ to confine NL-3 strain to the inoculated leaf.

**Table 6:** Phenotypic reactions to BCMNV NL-8 and NL-3 strains, pooled across F_2:3_ families from multiple crosses with the same F_2_ genotype based on SNP genetic markers linked with *I* (S02_48908259), *bc-1* (S03_4203361), and *bc-u*^d^ (Pvbzip1_A_C) genes.

Genotypes/Virus strain	F_2_ progenitor SNP markers *I/bc-1/bc-u*^d^	Number of F_2:3_ plants	Non *i* gene				*I* gene			Observed ratio	Probability *X*^2^ test; *α* = 0.05
			M	dM	mM	NS	TN	dTN or VN^+^	VN		
NL-8	^a^+/−/−	42					42			1:0	0.00
	+/−/+	118						118		0:1	0.00
	+/+/−	63							63	0:1	0.00
	+/+/+	122							122	0:1	0.00
	+/+/H	55							55	0:1	0.00
	+/H/+	80						25	55	1:3	1.67
	+/H/−	40					14		26	1:3	2.13
	+/−/H	39					31	8		3:1	0.42
	+/H/H	36					7	3	26	3:1:12	0.30
	H/+/+	16				3			13	1:3	0.33
	H/−/+	21		2				19		1:3	2.68
	−/H/+	18		4	10	4				1:2:1	0.22
	H/H/+	19		0	1	3		5	10	1:2:1:3:9	5.80
	**TOTAL**	**669**	**0**	**6**	**11**	**10**	**94**	**178**	**370**	**N/A**	**N/A**
NL-3	+/−/−	26					26			1:0	0.00
	+/−/+	109					109			1:0	0.00
	+/+/−	55					55			1:0	0.00
	+/+/+	117							117	0:1	0.00
	+/+/H	52					14	22	16	1:2:1	1.38
	+/H/+	76					23	29	24	1:2:1	4.29
	+/H/−	37					37			1:0	0.00
	+/−/H	36					36			1:0	0.00
	+/H/H	35					12	22	1	7:8:1	2.52
	H/+/+	18			8				10	1:3	3.63
	H/−/+	19	4				15			1:3	0.16
	−/H/+	15	3	9	3					1:2:1	0.60
	H/H/+	20	1	5	3		3	7	1	1:2:1:3:6:3	7.20
	**TOTAL**	**615**	**8**	**14**	**14**	**0**	**330**	**80**	**169**	**N/A**	**N/A**

a + and −, indicates homozygous for the resistance and susceptible marker alleles, respectively. H indicates heterozygotes.

#### Segregating Families: NL-8 Reactions

The F_2:3_ families fixed for *I* gene and *bc-1* genes (*II/bc-1bc-1*) all exhibited VN to NL-8 regardless of the allelic state for *bc-u*^d^. When the *I* gene was fixed and *bc-1* was segregating, the observed phenotypes fit a 1 susceptible (TN or dTN) to 3 resistant (VN) ratio. The resistant VN reaction for *II/Bc-1bc-1* heterozygotes was unexpected. Delayed TN by 1 week (dTN) was the susceptible reaction when *bc-u*^d^ was fixed (*II*/*bc-u*^d^*bc-u*^d^) and TN when (*II*/*Bc-u*^d^*Bc-u*^d^) or heterozygous (*II*/*Bc-u*^d^*bc-u*^d^) were absent. When both *bc-u* and *bc-1* were segregating and the *I* gene was fixed a dihybrid 3(TN):1(dTN):12(VN) segregation ratio was observed, which fit with the segregation ratios observed above for each gene.

The segregation of *bc-1* in an F_2:3_ family with no *I* gene and fixed for *bc-u*^d^ exhibited a 1(dM):2(mM):1(NS) segregation ratio for reaction to NL-8. The heterozygous *ii/Bc-1bc-1/bc-u*^d^*bc-u*^d^ individuals had partial mM resistance. In the absence of *I* or *bc-1*, individual homozygous for *bc-u*^d^ (*ii/Bc-1Bc-1/bc-u*^d^*bc-u*^d^) had mosaic symptoms delayed (dM) by 1 week, whereas individual homozygous for both *bc-1* and *bc-u*^d^ (*ii/bc-1bc-1/bc-u*^d^*bc-u*^d^) had no symptoms.

#### Segregating Families: NL-3 Reactions

The F_2:3_ families fixed for *I* and *bc-1* genes (*II/bc-1bc-1*) and segregating for *bc-u*^d^, exhibited 1(TN):2(dTN):1VN phenotypic segregation ratio to NL-3 strain. In this case, the heterozygous *II/bc-1bc-1/Bc-u*^d^*bc-u*^d^ genotype exhibited dTN, and homozygous genotypes *II/bc-1bc-1/Bc-u*^d^*Bc-u*^d^ and *II/bc-1bc-1/bc-u*^d^*bc-u*^d^ had TN and VN, respectively. For F_2:3_ families with *I* and *bc-u*^d^ genes fixed (*II/bc-u*^d^*bc-u*^d^) and *bc-1* segregating a 1(TN):2(VN^+^):1(VN) phenotypic segregation ratio was observed with most heterozygous genotypes for *bc-1* (*II/Bc-1bc-1/bc-u*^d^*bc-u*^d^) exhibiting VN^+^ (48% of plants) or dTN (33%) and a few VN (19%). For these *II/Bc-1bc-1/bc-u*^d^*bc-u*^d^ genotypes, VN^+^ and dTN symptoms started to appear in some plants at 3 wpi (Supplementary Table S3). The variable reactions for the heterozygotes may result from slight differences in virus titer or environmental conditions between inoculations. The homozygous individuals *II/Bc-1Bc-1/bc-u*^d^*bc-u*^d^ and *II/bc-1bc-1/bc-u*^d^*bc-u*^d^ had TN and VN, respectively.

The segregation of *bc-1* in an F_2:3_ family with no *I* gene and fixed for *bc-u*^d^ exhibited a 1(M):2(dM):1(mM) segregation ratio for reaction to NL-3. The heterozygous individuals *ii/Bc-1bc-1/bc-u*^d^*bc-u*^d^ had mosaic symptoms delayed by 1 week (dM). The homozygous *ii/Bc-1Bc-1/bc-u*^d^*bc-u*^d^ and *ii/bc-1bc-1/bc-u*^d^*bc-u*^d^ had M and mM, respectively.

## Discussion

Genome-wide association study of the DDP confirmed the *Bc-1* locus on Pv03 and located the *Bc-u* locus on Pv05. High-density SNP genotyping of the DDP using WGS enabled GWAS to detect narrow peak intervals for both loci and facilitate the discovery of candidate genes. The candidate gene markers developed for both loci were used to examine the phenotypic segregations in F_2_ populations and select F_2:3_ families. The candidate gene markers for *bc-1* and *bc-u*^d^ (formerly *bc-u*) and Tm-shift primers developed for *bc-3* and the new marker for *I* gene, were further used to examine the resistance genotypes in the DDP and HG differentials. Ultimately, the marker for *bc-u*^d^ was key for the discovery of major adjustments to the host-pathogen model from Drijfhout (1978) seminal work on pathogenicity and resistance to BCMNV/BCMV in common bean.

First, we show that there is only one recessive resistance allele *bc-1* for the *Bc-1* locus. This finding is supported by disease reaction and molecular marker analyses in the segregating populations and assays of the DDP and host differential cultivars. Furthermore, sequencing data for the RLK candidate gene exons did not detect any polymorphic variants between *bc-1* (RGC) and *bc-1*^2^ (Olathe) genotypes. So, instead of two alleles (*bc-1* and *bc-1*^2^), the differential expression for *bc-1* results from the absence vs. presence of the *bc-u*^d^ resistance allele.

Another new finding for *bc-1* is the dominant and incompletely dominant inheritance observed in F_2:3_ families. Heterozygous *Bc-1bc-1* and homozygous *bc-1bc-1* genotypes with *I* gene fixed exhibited dominant VN symptoms to NL-8. *Bc-1bc-1* heterozygotes with *I* and *bc-u*^d^ genes fixed exhibited incomplete dominance (VN^+^) to NL-3 strain, which for most testing scenarios would reflect dominant inheritance because the vein necrosis reaction does not appear systemically until 3–5 wpi. Miklas et al. (2000) tagged *bc-1*^2^ with the SBD5 SCAR marker using near-isogenic lines (NILs) they thought differed for *bc-1* alleles (*bc-1* vs. *bc-1*^2^). They reported the order of dominance as *bc-1*^2^>*bc-1*. However, in retrospect, each of their NILs possessed the dominant *I* and recessive *bc-u*^d^ genes but differed for presence vs. absence of *bc-1*. This explains the dominant inheritance for resistance to NL-3 observed in an F_2_ population obtained from a cross between the NILs.

We also observed incomplete dominance for *bc-1* in the absence of *I* gene (*ii* genotypes) and homozygous presence of *bc-u*^d^, but in this case, the disease reactions for the *Bc-1bc-1* heterozygotes, mM to NL-8 and dM to NL-3, more closely resembled the homozygous (*Bc-1Bc-1*) susceptible dM and M reactions, respectively. We were unable to examine the inheritance of *bc-1* in the absence of *I* and *bc-u*^d^ genes because our crosses did not segregate for those genotypes. However, DDP lines (“Sapphire” and “Croissant”) with markers indicating the presence of the recessive *bc-1* only were resistant (NS) to NL-8 and susceptible (M) to NL-3, indicating *bc-1* by itself is effective in conditioning resistance against some BCMNV strains.

A second major change to the host-pathogen interaction model is that *bc-u* is not ubiquitous across HGs 2–7, which lack the *I* gene. This new finding necessitated the need to mark this absence of *bc-u* in HGs where it was previously thought to be present. Therefore, we added the “d” superscript to track this specific allele going forward. The HGs 2, 4, 5, and 7 lacked *bc-u*^d^, whereas *bc-u*^d^ was present in HG-6 and most HG-3 genotypes. For HGs with *I* gene, *bc-u*^d^ was absent in HG-8 and HG-9a and 9b and present in HG-11 as expected, but it was also present in some HG-10 genotypes, which was unexpected.

All DDP lines in HG-3 and HG-10 possessed *bc-u*^d^, whereas *bc-u*^d^ is absent in many snap beans or lines of Andean origin within these HGs. Redlands Greenleaf B (RGB), an Andean line categorized as HG-3, and reported previously to possess the *bc-u* gene, showed the wild type *Bc-u* protein form. Strausbaugh et al. (2003) observed quantitative differences in levels of partial resistance among lines in HG-3 inoculated with NL-3, and we hypothesize that these quantitative differences are due to different/independent “*bc-u*”-like helper genes. A homologous bZIP domain gene Phvul.011G093700 on Pv11 (G19833 v2.1: 9,431,906–9,439,104 base pairs) with 73.9% similarity to Phvul.005G124100 is under investigation as an alternative helper gene for HG-3 lines with *bc-1*, such as RGB, and Amanda in HG-10.

Further investigation of why some HG-1 differentials, such as Bountiful and stringless green refugee (SGR), that are susceptible to all BCMNV and BCMV strains possess the *bc-1* linked marker, whereas others, like Dubbele Witte and Sutter Pink, do not, is warranted. Myers et al. (1996) observed segregation for susceptible and resistant reactions in an F_2_ cross between Sutter Pink (susceptible) and SGR (resistant) inoculated with BCMV strain *Blackeye cowpea mosaic virus* (BCMV-BlCMV). A single resistance gene from SGR with dominant inheritance was observed when symptoms were recorded 3 wpi and with incomplete dominance when recorded 7 wpi. They indicated SGR possessed *bc-u* and perhaps *bc-u* or some other genetic factor was conferring resistance to BCMV-BlCMV. The marker results reveal that SGR possesses *bc-1* but not *bc-u*. However, we similarly observed dominant gene action at 3 wpi and partial dominance at 5 wpi to BCMNV for some F_2:3_ families segregating for *bc-1*. Larsen and Miklas (2010) observed *bc-1* conferred resistance to *Peanut mottle virus* (PeMoV), and in subsequent F_2_ populations (unpublished data) observed dominant gene action.

*bc-u* (now *bc-u*^d^) is relocated on Pv05, and independent from *bc-1*, contrary to the loose (23 cM) linkage observed between them by Strausbaugh et al. (1999). Perhaps the loose linkage was observed because some of the RILs were either misclassified as partially resistant to NL-3 when in fact they were susceptible or vice versa. They classified RILs with *Bc-uBc-u*/*bc-1*^2^*bc-1*^2^ genotype as expressing mM reactions, whereas we observed such genotypes to have susceptible M reactions to NL-3 in this study. These differences may be influenced by recording disease reactions every week for 5 wpi, whereas they recorded disease reactions once at 3 wpi.

Another new finding for *bc-u*^d^ is that it conditioned a delay in the onset of susceptible reactions by about 1 wpi when *bc-1* was absent and significantly enhanced the level of resistance in the presence of *bc-1*. *Bc-1Bc-1/bc-u*^d^*bc-u*^d^ genotypes inoculated with NL-8 exhibited dTN and dM symptoms in the presence vs. absence of *I* gene, respectively, whereas *bc-1bc-1/bc-u*^d^*bc-u*^d^ genotypes inoculated with NL-3 exhibited resistant VN and partially resistant mM symptoms. Moreover, *II/bc-1bc-1/Bc-u*^d^*bc-u*^d^ genotype exhibited dTN to NL-3, indicating *bc-u*^d^ in heterozygous condition with both *I* and *bc-1* genes fixed delayed the onset of TN by about 1 wpi.

Plants with the *I* gene can be useful for understanding the mechanisms of other recessive resistance genes (*bc-u*^d^, *bc-1*) when present in combination with the *I* gene. The *I* gene alone conditions HR to BCMNV strains in common bean leading to TN and eventual plant death. This TN symptom in beans is similar to the phenomenon known as trailing necrosis, where HR does not prevent the pathogen from moving systemically from cell to cell, and in turn expands the HR to other cells and tissues (Balint-Kurti, 2019). Although HR is associated with pathogen resistance, when it results in systemic cell to cell movement and eventual plant death, it can be considered a consequence of pathogen compatibility. Whether the HR mechanism leads to systemic cell death (TN), delayed TN, or restricted vein necrosis (VN) is controlled by other proteins that recognize specific pathogen effectors, such as receptor molecules like RLK and NBS-LRR proteins. Moreover, several families of transcription factors have been discovered to regulate programmed cell death and HR, including members of bZIP, MBY, NAC, ERF, and WRKY (Burke et al., 2020).

In the present study, we identified a candidate gene for *bc-u*^d^ that encodes a bZIP transcription factor. This candidate gene (Phvul.005G124100) is located on Pv05 from 36,113,780 to 36,120,803 bases. The results for *bc-u*^d^ predict a loss-function of bZIP protein due to the presence of a stop-gain variant. The silenced protein combined with the *I* and *bc-1* genes affects complete resistance characterized by restricted vein necrosis on the inoculated primary leaves to BCMNV strain NL-3. Several studies have demonstrated the importance of bZIP transcription factors modulating different regulatory networks and signaling pathways related to physiological processes (Alves et al., 2013). Some of these processes are related to biotic stress, particularly in the defense against pathogens. In *Arabidopsis thaliana*, the bZIP protein TGA9 (AT1G08320), which regulates the autophagy under stress conditions, showed in TGA9–2 and TGA9–3 mutants, significative repression of autophagy activation under stress but not completely blocked (Wang et al., 2020). Another bZIP in *A. thaliana*, AtbZIP10, has been identified as a positive regulator of the pathogen-induced hypersensitive cell-death response (HR) and in basal defense response. AtbZIP10 interacts with LSD1 (lesions simulating disease resistance 1), a protein that protects against cell death mediated by oxidative stress signals (Kaminaka et al., 2006). Genetic interactions have been reported between bZIP transcription factors and the tobacco (*Nicotiana tabacum*) NPR1 gene, an NBS-LRR class protein conferring resistance to Tobacco mosaic virus (TMV; Liu et al., 2002). When only *bc-u*^d^ is combined with the *I* gene, the HR response is delayed by about 1 week in response to NL-8.

Receptor-like protein kinases play a relevant role in interaction networks between plants and viruses (Tang et al., 2017; Macho and Lozano-Duran, 2019). Proteins with receptor-like domains interact with viral movement proteins promoting the viral movement into tubules within plasmodesmata (Amari et al., 2010). The *bc-1* recessive gene was found to affect the systemic spread of BCMV in common beans (Feng et al., 2017, 2018). We identified two RLKs as candidate genes for *bc-1* in this study. The two RLK genes on Pv03 in the common bean are orthologous with two RLK genes on Gm02 in soybean reported separately to condition resistance to TRSV and SMV (Ilut et al., 2015; Chang et al., 2016). Both RLK genes were identified as candidate genes for the *Rsv4* gene, which presents a dominant or semi-dominant resistance to SMV in soybean (Ilut et al., 2015). Another study in soybean revealed a major QTL near *Rsv4*, which conditioned resistance to Clover yellow vein virus (ClYVV). This QTL was named *d-cv* gene to acknowledge the recessive inheritance as different from the *Rsv4* gene (Abe et al., 2019). In summary, the synteny analysis between common bean and soybean shows that the genomic regions possessing *bc-1* and *Rsv4* genes are orthologous and involved in resistance to multiple ssRNA (+) viruses. Finally, Phvul.003G039000, which encodes a MIF4G domain-containing protein, cannot be discounted as a candidate gene for *bc-1*. However, a MIF4G annotated gene is absent from the orthologous *Rsv4* region on Gm02.

Many breeding programs are moving toward the introgression of the *bc-3* (eIF4e) resistance gene to control BCMNV and BCMV. However, a new BCMV isolate has been identified, 1755a (PG-VIII), which causes mosaic and deformed leaf symptoms in some common bean lines with the *bc-3* gene (Feng et al., 2015). This study shows that *bc-u*^d^ and *bc-1* can be introgressed in breeding lines to achieve suitable and improved resistance to BCMNV. Perhaps, *bc-u*^d^ combined with *bc-3* will provide resistance to the new BCMV strain. Deploying *bc-u*^d^ in a snap and Andean beans that are nearly ubiquitous for *bc-1* would broaden the virus resistance of those market classes and gene pools. The SNP markers developed will be useful for deploying *bc-u*^d^ and *bc-1* resistance genes in common bean, and those tools combined with the identified candidate genes will help to further elucidate the host-pathogen interaction between potyviruses and common bean.

## Data Availability Statement

The original contributions presented in the study are publicly available. This data can be found here: NCBI SRA database with accession Bioproject PRJNA386820.

## Author Contributions

AS-G and PNM conceived and designed the experiments, and wrote the paper. AS-G conducted the genomics analyses, genotyping, and phenotyping. PEM generated SNP data. All authors contributed to the article and approved the submitted version.

### Conflict of Interest

The authors declare that the research was conducted in the absence of any commercial or financial relationships that could be construed as a potential conflict of interest.

## Footnotes

^1^https://www.ncbi.nlm.nih.gov/bioproject/?term=PRJNA386820

^2^https://phytozome-next.jgi.doe.gov/info/Pvulgaris_v2_1

^3^https://phytozome-next.jgi.doe.gov/info/Gmax_Wm82_a4_v1
