# Supplementary material for: Genome-Wide Association Mapping of bc-1 and bc-u Reveals Candidate Genes and New Adjustments to the Host-Pathogen Interaction for Resistance to Bean Common Mosaic Necrosis Virus in Common Bean
Source: Front Plant Sci. 2021 Jun 29;12:699569. doi: 10.3389/fpls.2021.699569 (PMC8277298; doi:10.3389/fpls.2021.699569)
Supplement: Supplementary file 1 [file Presentation_1.pptx]

## Slide 1
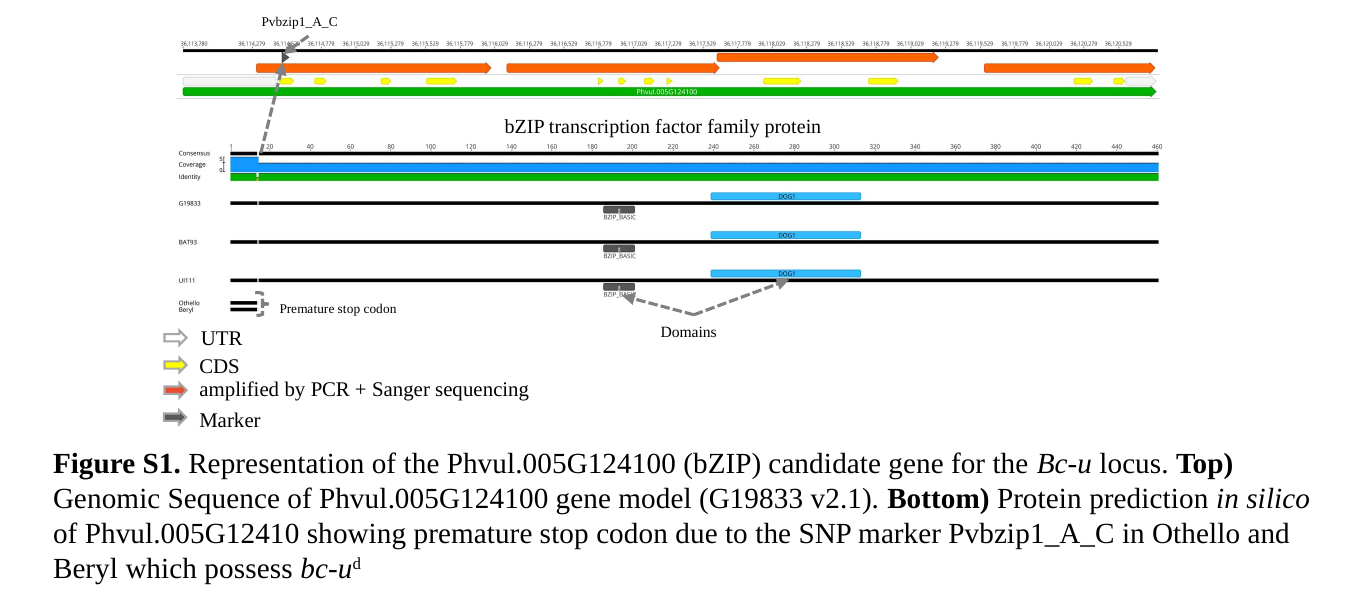

Pvbzip1_A_C
bZIP transcription factor family protein
Premature stop codon
Domains
UTR
CDS
amplified by PCR + Sanger sequencing
Marker
Figure S1. Representation of the Phvul.005G124100 (bZIP) candidate gene for the Bc-u locus. Top) Genomic Sequence of Phvul.005G124100 gene model (G19833 v2.1). Bottom) Protein prediction in silico of Phvul.005G12410 showing premature stop codon due to the SNP marker Pvbzip1_A_C in Othello and Beryl which possess bc-ud

## Slide 2
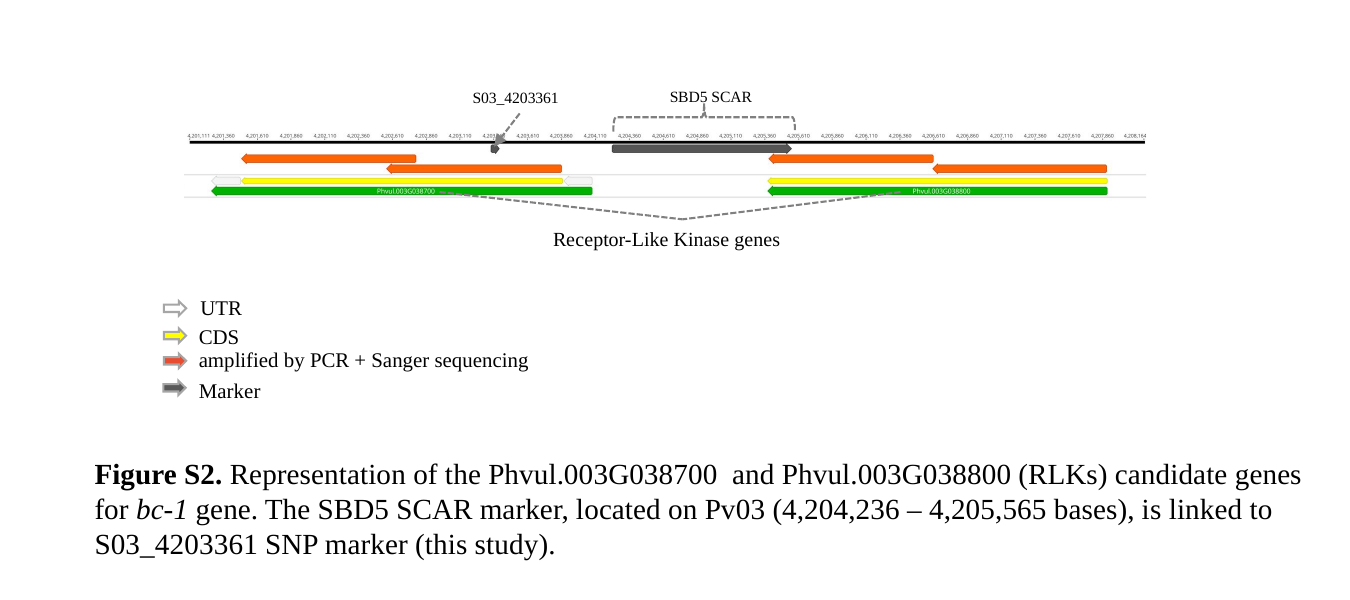

SBD5 SCAR
S03_4203361
Receptor-Like Kinase genes
UTR
CDS
amplified by PCR + Sanger sequencing
Marker
Figure S2. Representation of the Phvul.003G038700 and Phvul.003G038800 (RLKs) candidate genes for bc-1 gene. The SBD5 SCAR marker, located on Pv03 (4,204,236 – 4,205,565 bases), is linked to S03_4203361 SNP marker (this study).

## Slide 3
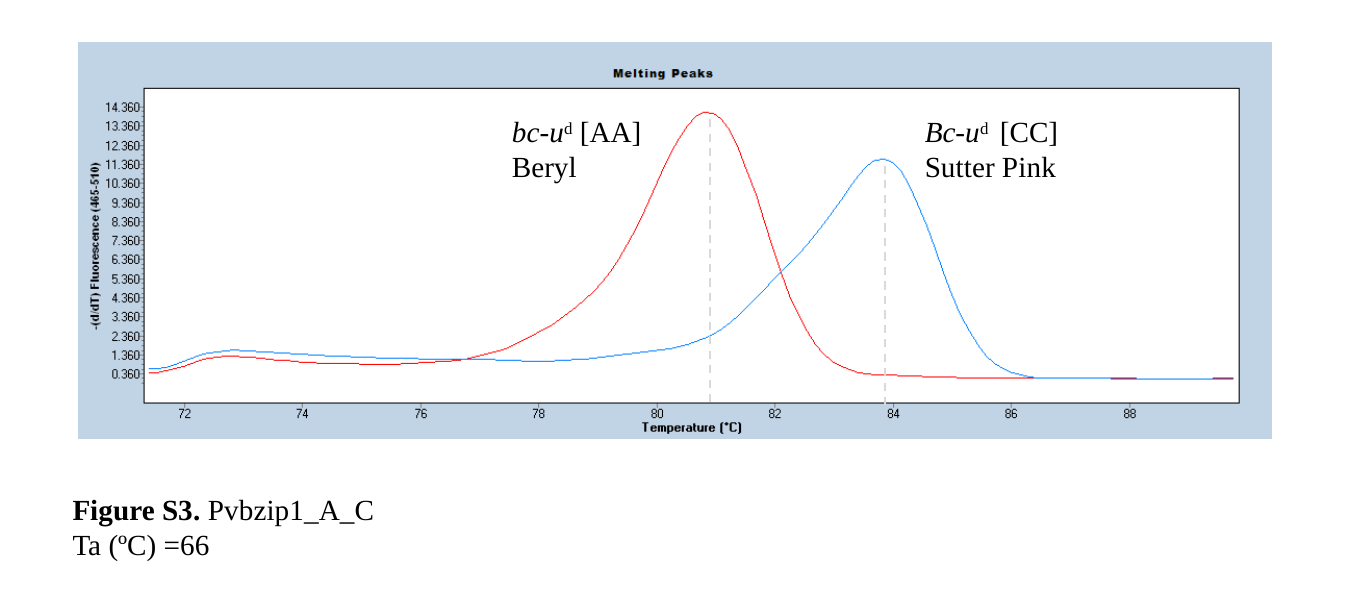

bc-ud [AA]
Beryl
Bc-ud [CC]
Sutter Pink
Figure S3. Pvbzip1_A_C
Ta (ºC) =66

## Slide 4
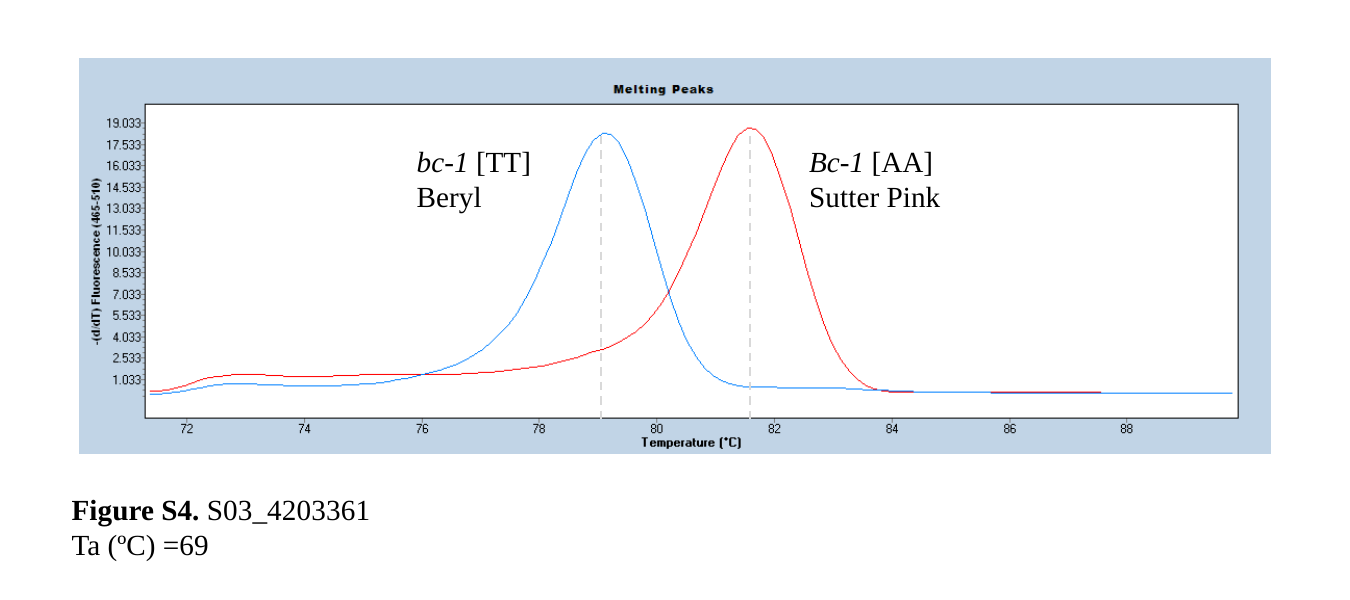

bc-1 [TT]
Beryl
Bc-1 [AA]
Sutter Pink
Figure S4. S03_4203361
Ta (ºC) =69

## Slide 5
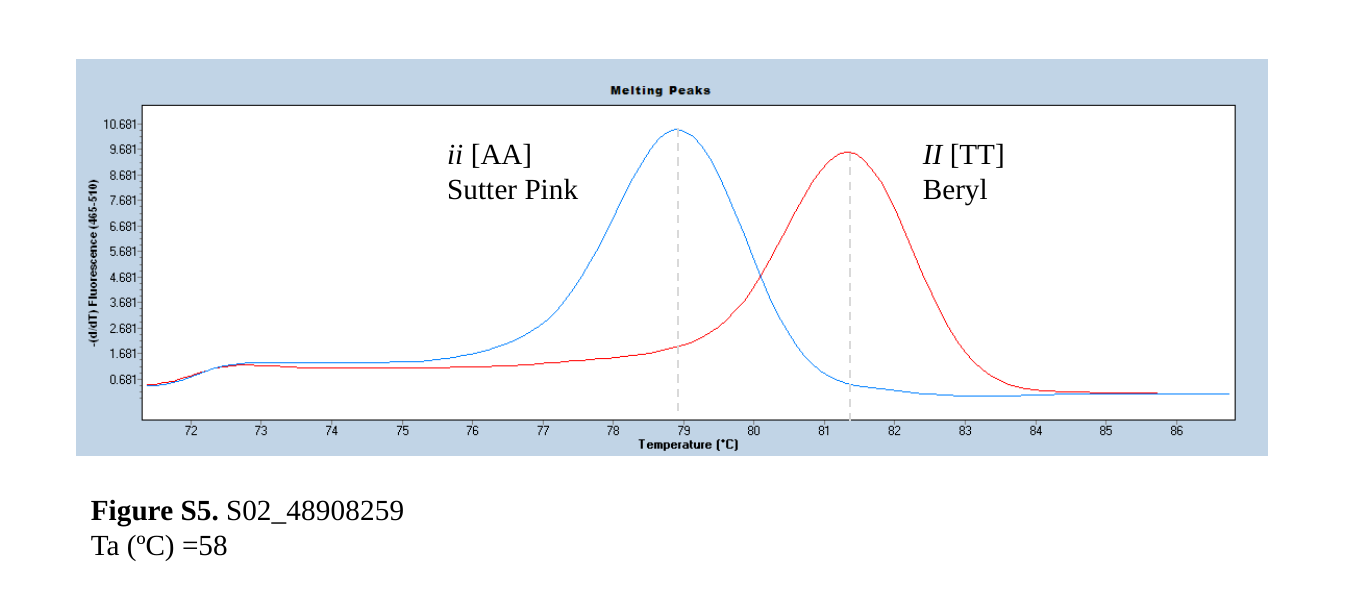

ii [AA]
Sutter Pink
II [TT]
Beryl
Figure S5. S02_48908259
Ta (ºC) =58

## Slide 6
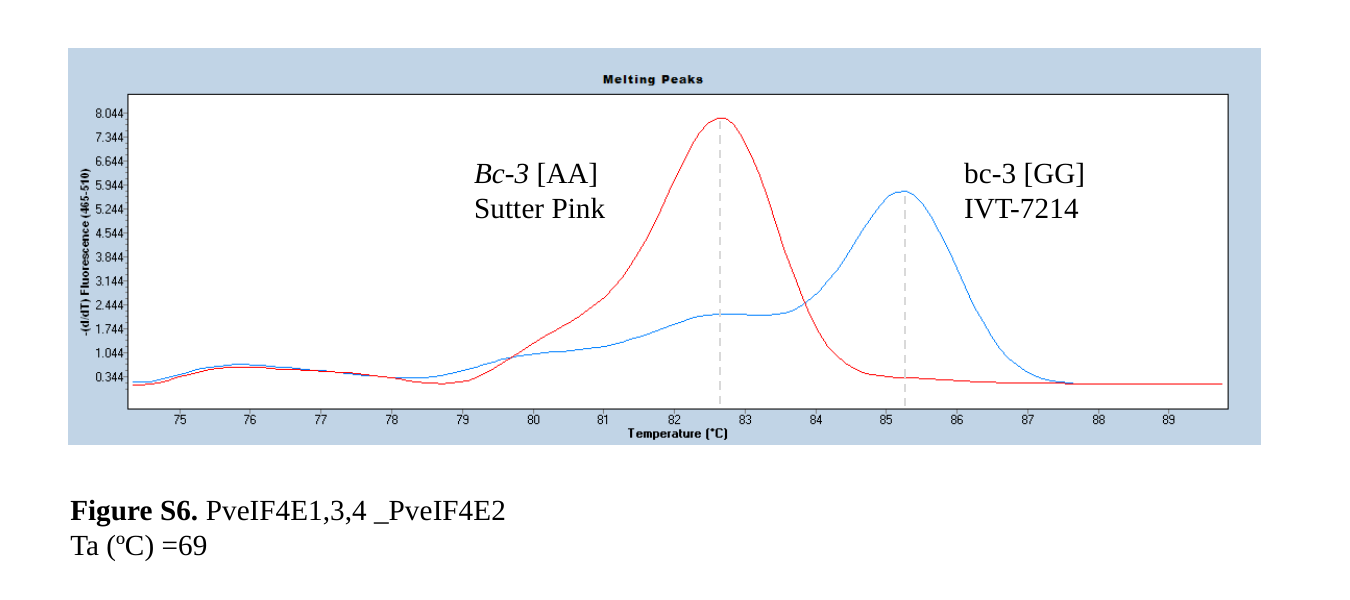

Bc-3 [AA]
Sutter Pink
bc-3 [GG]
IVT-7214
Figure S6. PveIF4E1,3,4 _PveIF4E2
Ta (ºC) =69
