# Supplementary figures and images for: Genome-Wide Association Mapping of bc-1 and bc-u Reveals Candidate Genes and New Adjustments to the Host-Pathogen Interaction for Resistance to Bean Common Mosaic Necrosis Virus in Common Bean
Source: Front Plant Sci. 2021 Jun 29;12:699569. doi: 10.3389/fpls.2021.699569 (PMC8277298; doi:10.3389/fpls.2021.699569)

Pvbzip1\_A\_C

Ta (°C) =66

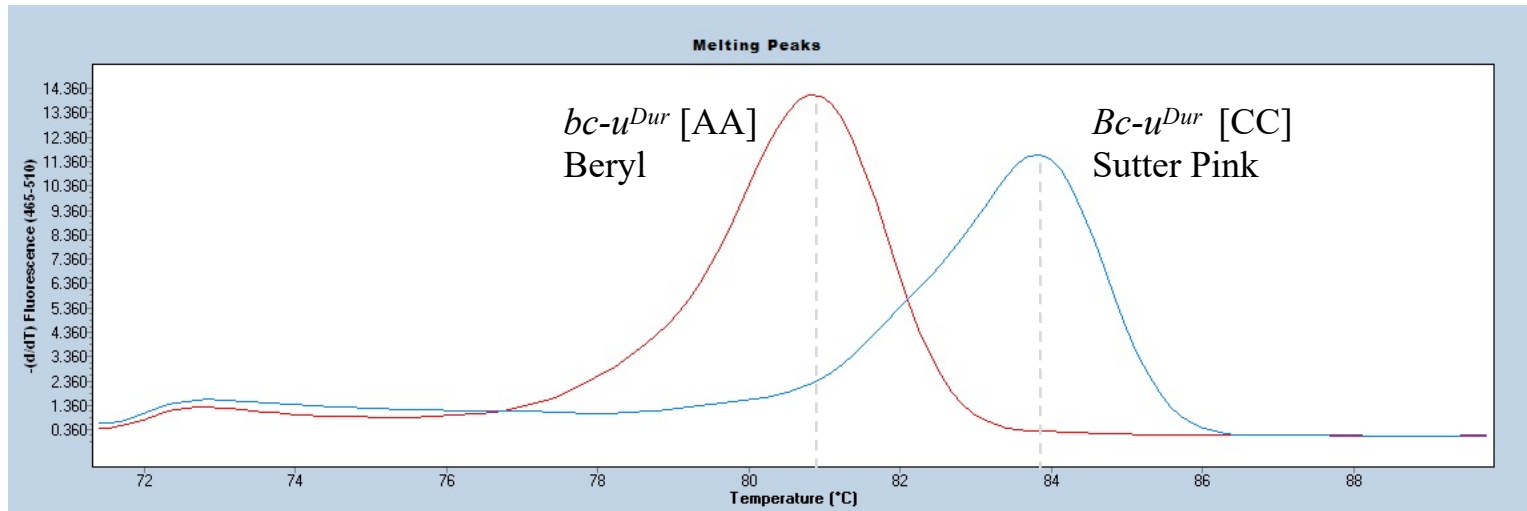

S03\_4203361

Ta (°C) =69

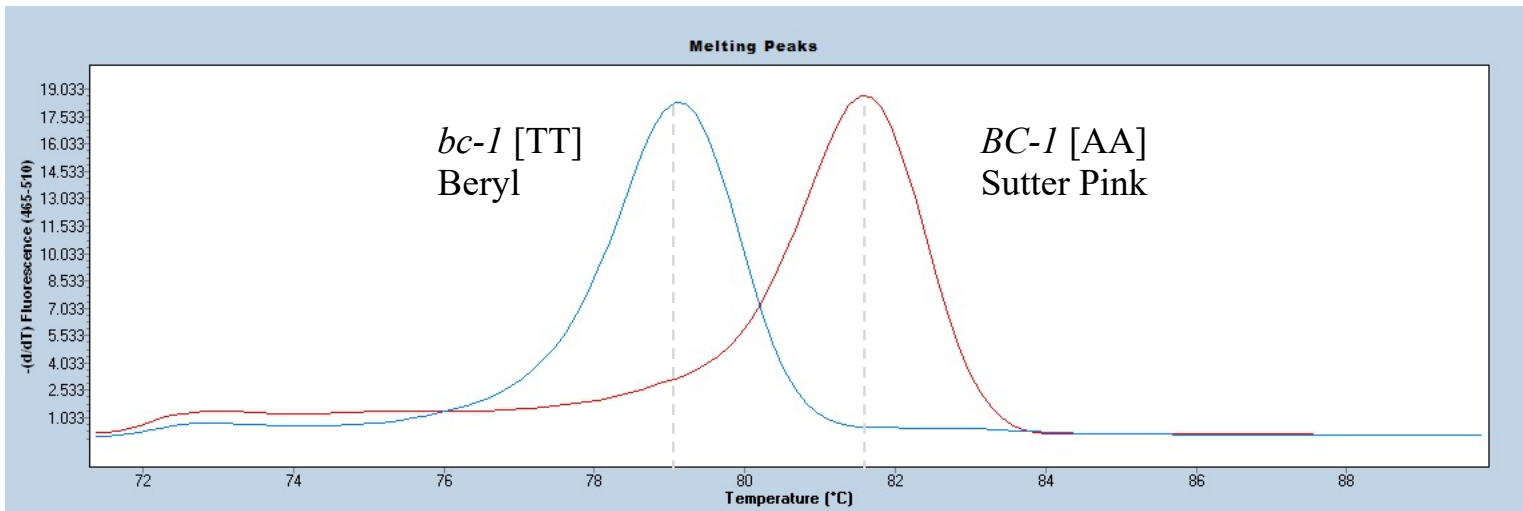

S02\_48908259

Ta (°C) = 58

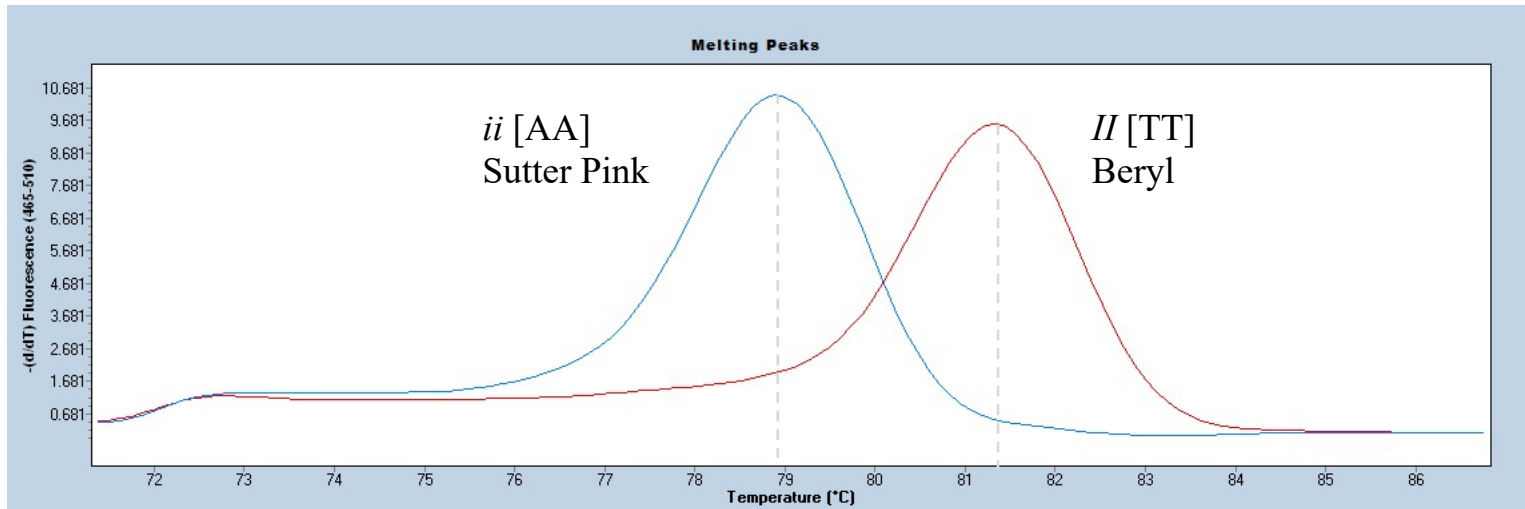

PveIF4E1,3,4 \_PveIF4E2

Ta (°C) =69

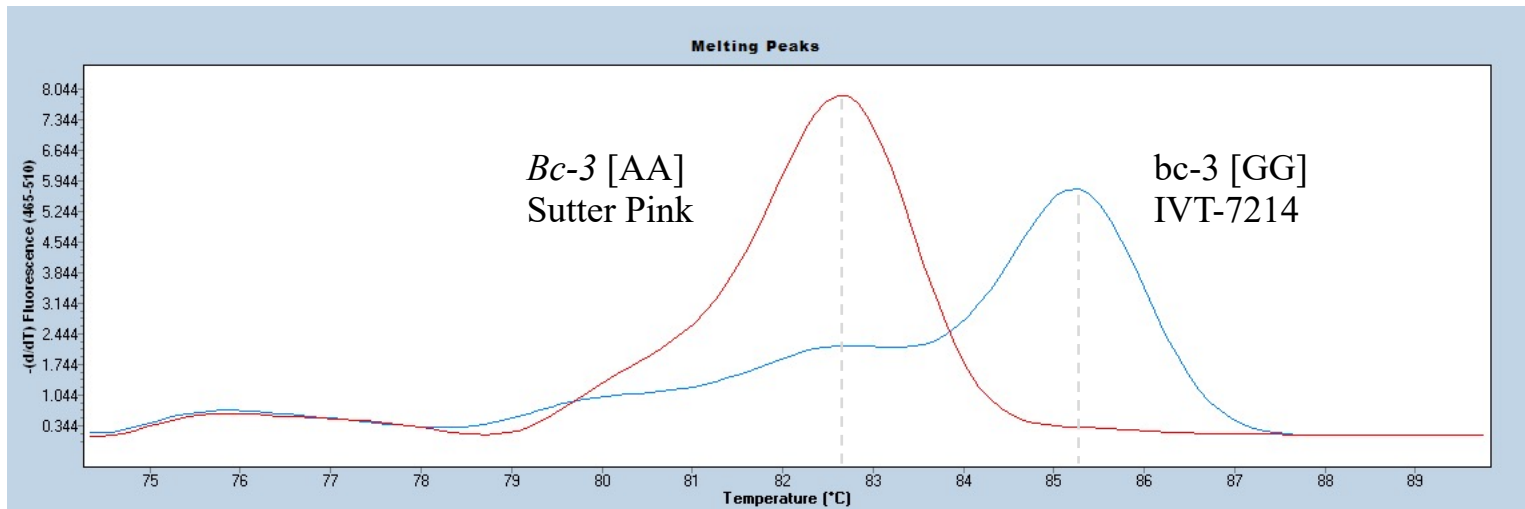

Supplement: Supplementary file 2 [file Data_Sheet_1.PDF]
